# Butyrate enhances tumor immune evasion by increasing PD-L1 abundance

**DOI:** 10.7150/ijbs.131184

**Published:** 2026-07-20

**Authors:** Xue Sun, Yi Gao, Jiaxi Li, Yan Ma, Hongchen Li, Zhen Wang, Lei Lv, Yanping Xu

**Affiliations:** 1 Tongji Hospital, Frontier Science Center for Stem Cell Research, Shanghai Key Laboratory of Signaling and Disease Research, School of Life Sciences and Technology, Tongji University, Shanghai, China.; 2 Nourse Centre for Pet Nutrition, Wuhu, China.; 3 Zhejiang Provincial Engineering Research Center of New Technologies and Applications for Targeted Therapy of Major Diseases, Zhejiang Provincial Key Laboratory of Drug Discovery and Development for Metabolic Diseases, College of Life Sciences and Medicine, Zhejiang Sci-Tech University, Hangzhou, Zhejiang Province, China.; 4 MOE Key Laboratory of Metabolism and Molecular Medicine, Department of Biochemistry and Molecular Biology, School of Basic Medical Sciences, Fudan University, Shanghai, China.; 5 Hebei University of Engineering, School of Medicine, Handan, Hebei, China.

**Keywords:** butyrate, PD-L1, TGM2, high-fiber diets

## Abstract

As a short-chain fatty acid and histone deacetylase (HDAC) inhibitor, butyrate possesses extensive immunomodulatory properties. Here, we uncover a novel mechanism wherein butyrate markedly upregulates the expression of the immune checkpoint PD-L1 across diverse tumor cell types. Mechanistically, epigenetic analyses revealed that butyrate drives PD-L1 transcriptional activation through dual synergistic pathways: increasing histone H3 acetylation (H3Ac) at the *PD-L1* promoter via HDAC inhibition, and enhancing histone H3K4me3 modification by upregulating transglutaminase 2 (TGM2). Combining butyrate or high-fiber diets with anti-PD-L1 antibodies profoundly suppressed tumor growth and bolstered anti-tumor immunity in both subcutaneous and AOM-DSS-induced colorectal cancer (CRC) murine models. Ultimately, our results highlight butyrate and dietary fiber supplementation as promising sensitizing strategies to augment the efficacy of PD-L1 blockade in patients with low baseline PD-L1 expression.

## Introduction

Butyrate, a four-carbon short-chain fatty acid (SCFA), is produced by gut microbiota through the fermentation of dietary fiber. Although accounting for approximately 15% of total human SCFAs, butyrate has garnered significant interest due to its beneficial roles in cellular energy metabolism and intestinal homeostasis^1^. It is well established that many of its biological effects are mediated through histone deacetylase (HDAC) inhibition, which suppresses cell proliferation, induces differentiation and apoptosis, and regulates gene expression^2^, ^3^. Accumulating evidence highlights butyrate's immunomodulatory capacity across diverse immune cell populations. Experimental studies demonstrate its regulatory effects on dendritic cells^4^, ^5^, macrophages^6^, B cells^7^, and T cells^8^, ^9^, collectively supporting intestinal immune homeostasis. Notably, recent findings indicate that butyrate directly enhances the IL-12 signaling pathway in an ID2-dependent manner, potentiating CD8^+^ T cell anti-tumor cytotoxicity^10^. Nevertheless, whether butyrate influences immunotherapy efficacy by modulating tumor cells, particularly the expression of immune checkpoints, remains unclear.

Programmed death-ligand 1 (PD-L1) is a well-characterized immune checkpoint molecule that inhibits T cell cytotoxicity by binding programmed cell death protein 1 (PD-1), thereby facilitating tumor immune evasion. Immune checkpoint blockade (ICB) therapy targeting the PD-L1/PD-1 axis has achieved considerable success in oncology^11^, ^12^. However, a substantial proportion of patients remain unresponsive to ICB^13^, ^14^. Studies suggest that this resistance may correlate with the abundance of immune checkpoints molecules on tumor cells^15^, as high tumor PD-L1 expression is often associated with increased T cell infiltration and improved response rates to anti-PD-1/PD-L1 therapy^16^, ^17^. Supporting this paradigm, research has shown that CMTM4/6 stabilizes PD-L1 by inhibiting STUB1-mediated polyubiquitination, which may improve the effectiveness of current PD-L1/PD-1 blocking therapy^18^. Consistent with the above viewpoint, some studies have classified tumors into four groups based on the abundance of PD-L1 and the presence of tumor-infiltrating lymphocytes (TIL), among which type I cancers (PD-L1^+^ TIL^+^) are considered to be most likely to benefit from anti-PD-L1 antibodies in clinical practice^19^, ^20^. Therefore, identifying agents capable of augmenting PD-L1 expression in tumor cells represents a potential strategy to improve response rates to anti-PD-1/PD-L1 therapy in patients with low baseline tumor PD-L1 expression.

In this study, we demonstrate that butyrate markedly upregulates PD-L1 expression across various tumor cell types. Mechanistically, butyrate enhances PD-L1 transcription through two distinct pathways: 1) increasing histone H3 acetylation (H3Ac) in the PD-L1 promoter region; 2) upregulating TGM2, which promotes H3K4me3 at the PD-L1 promoter, further driving its transcription. Critically, combining butyrate with anti-PD-L1 antibodies effectively suppressed subcutaneous tumor growth in mice. Similarly, high-fiber diets, which promote endogenous butyrate production through gut microbial fermentation, synergized with anti-PD-L1 therapy to inhibit AOM-DSS-induced colorectal cancer progression. Our findings reveal a novel epigenetic mechanism by which butyrate enhances tumor immunotherapy and establish that butyrate supplementation or high-fiber diets can significantly improve the efficacy of PD-L1 blockade. These results highlight the potential of dietary butyrate modulation as a strategy to sensitize low PD-L1-expressing tumors to immunotherapy, offering a promising approach to enhance treatment responses in cancer patients.

## Results

### Butyrate reduces T cell-mediated tumor cell killing through upregulation of PD-L1

**Figure 1:**
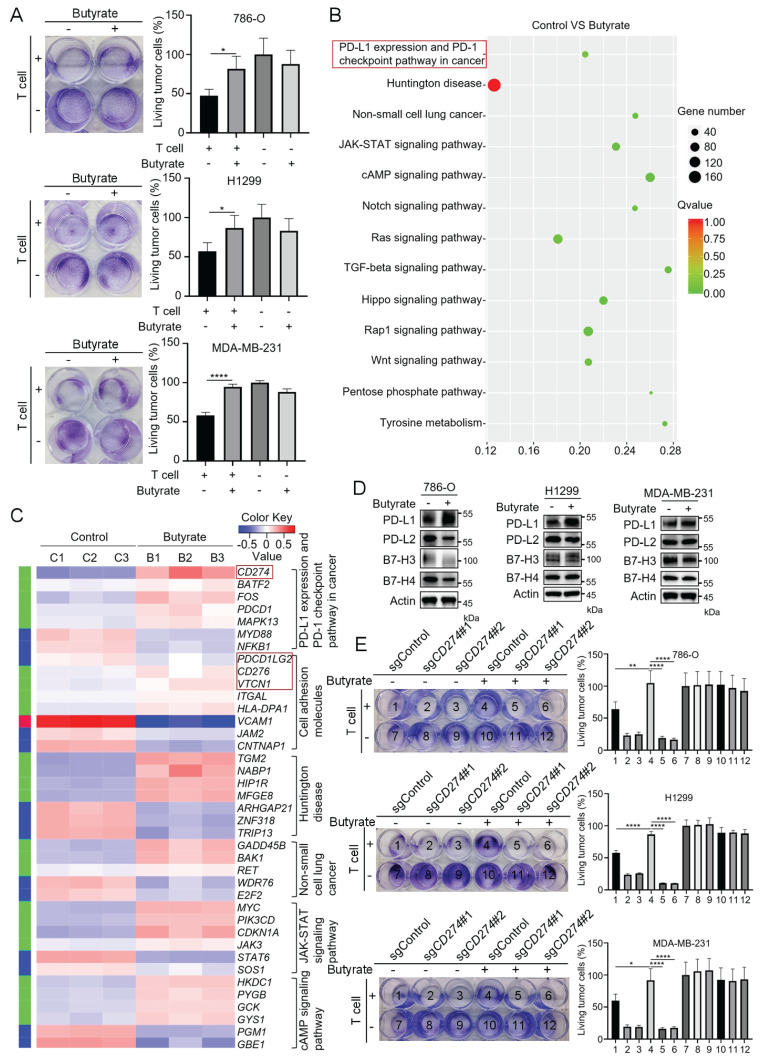
** Butyrate reduces T cell-mediated tumor cell killing through upregulation of PD-L1.** (**A**) The killing effect of T cells on tumor cells (pre-treated with butyrate for 48 h) was measured. Tumor cells were co-cultured with activated T cells for 48 h, and the survival rate of tumor cells was detected by crystal violet staining. Values are means ± SD from n = 3 independent experiments. Statistical differences were determined by Student's *t* test. *P < 0.05, ****P < 0.0001. (**B**) RNA-seq analysis was performed on 786-O cells with and without butyrate (4 mM) treatment, using three biologically independent samples per group. (**C**) Heatmap showing the relative levels of selected genes based on the RNA-seq data. (**D**) Western blot analysis of PD-L1, PD-L2, B7-H3, and B7-H4 protein levels in 786-O, H1299, and MDA-MB-231 cells treated with butyrate (4 mM) for 48 h. (**E**) T cell-mediated cytotoxicity against tumor cells was assessed using wild-type (WT) and PD-L1-knockout (sg*CD274*) tumor cells pretreated with butyrate for 48 h. Tumor cells were co-cultured with activated T cells for 48 h, and tumor cell viability was assessed by crystal violet staining. Values are means ± SD from n = 3 independent experiments. Statistical differences were determined by Student's *t* test. *P < 0.05, **P < 0.01, ****P < 0.0001.

To evaluate the effect of butyrate on T cell-mediated tumor cell killing, we pretreated tumor cells with butyrate *in vitro* and co-cultured them with activated T cells (Fig. S1A). Strikingly, butyrate-pretreated tumor cells exhibited significantly higher survival than untreated controls (Fig. 1A), suggesting that butyrate impairs T cell-mediated tumor cell killing. To explore the pathway through which butyrate weakens the killing ability of T cells against tumor cells, we treated 786-O cells with butyrate and performed RNA sequencing (RNA-seq) analysis, noting that the “PD-L1 expression and PD-1 checkpoint pathway in cancer” was altered after butyrate treatment (Fig. 1B). Since immune checkpoint interactions between tumor and T cells can suppress T cell function, in addition to *CD274* (*PD-L1*), we also found that several other important immune checkpoints, such as *PDCD1LG2* (*PD-L2*), *CD276* (*B7-H3*), and *VTCN1* (*B7-H4*) were altered after butyrate treatment (Fig. 1C). To further confirm the RNA-seq results, we examined the expression of key checkpoint molecules (PD-L1, PD-L2, B7-H3, and B7-H4) in butyrate-treated 786-O, H1299, and MDA-MB-231 cells. Western blot analysis revealed that butyrate selectively upregulated PD-L1 while having no significant effect on other checkpoints (Fig. 1D), implying that PD-L1 induction may mediate butyrate's immunosuppressive effect.

To test this hypothesis, we generated a CRISPR/Cas9-mediated *PD-L1* knockout (sg*CD274*) tumor cell line (Fig. S1B) and performed T cell cytotoxicity assays. Crystal violet staining demonstrated that pretreatment with butyrate helps tumor cells resist T cell killing, whereas this protective effect was largely abolished in PD-L1-deficient cells (Fig. 1E). These results indicate that butyrate impairs T cell-mediated tumor cell killing, at least in part, through upregulation of PD-L1, suggesting a potential mechanism by which butyrate may contribute to tumor immune evasion.

**Figure 2:**
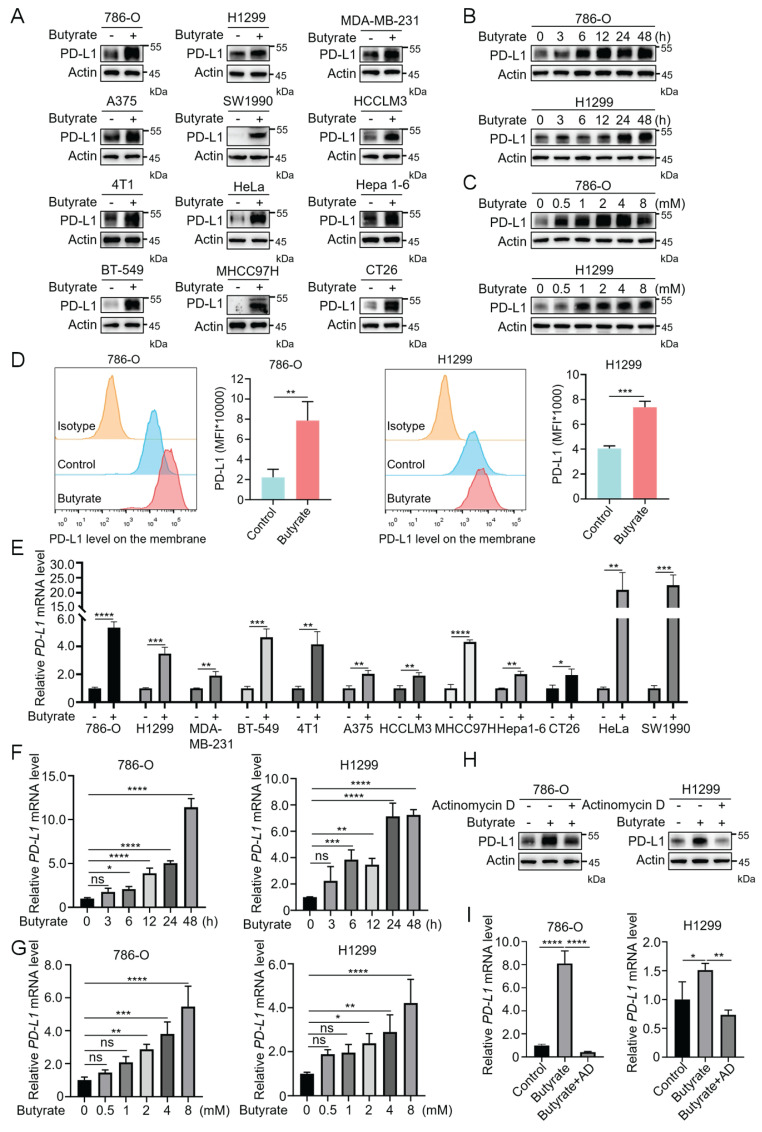
** Butyrate upregulates PD-L1 expression through transcriptional activation.** (**A**) Western blot analysis of PD-L1 protein levels in 786-O, H1299, MDA-MB-231, BT-549, 4T1, HeLa, A375, SW1990, HCCLM3, MHCC97H, Hepa 1-6, and CT26 cells treated with butyrate (4 mM) for 48 h. (**B**) Western blot analysis of PD-L1 protein levels in 786-O and H1299 cells treated with 4 mM butyrate for different times as indicated. (**C**) Western blot analysis of PD-L1 protein levels in 786-O and H1299 cells treated with different concentrations of butyrate as indicated for 48 h. (**D**) Flow cytometry analysis of membrane PD-L1 expression in 786-O and H1299 cells, and the summarized mean fluorescent intensity (MFI) is shown. Values are means ± SD from n = 3 independent experiments. Statistical differences were determined by Student's *t* test. **P < 0.01, ***P < 0.001. (**E**) qRT-PCR analysis of *PD-L1* levels in 786-O, H1299, MDA-MB-231, BT-549, 4T1, HeLa, A375, SW1990, HCCLM3, MHCC97H, Hepa 1-6, and CT26 cells treated with 4 mM butyrate for 48 h. Values are means ± SD from n = 3 independent experiments. Statistical differences were determined by Student's *t* test. *P < 0.05, **P < 0.01, ***P < 0.001, ****P < 0.0001. (**F**) qRT-PCR analysis of *PD-L1* levels in 786-O and H1299 cells treated with 4 mM butyrate for different times as indicated. Values are means ± SD from n = 3 independent experiments. Statistical differences were determined by ordinary one-way ANOVA. ns, nonsignificant, *P < 0.05, **P < 0.01, ***P < 0.001, ****P < 0.0001. (**G**) qRT-PCR analysis of *PD-L1* levels in 786-O and H1299 cells treated with different concentrations of butyrate as indicated for 48 h. Values are means ± SD from n = 3 independent experiments. Statistical differences were determined by ordinary one-way ANOVA. ns, nonsignificant, *P < 0.05, **P < 0.01, ***P < 0.001, ****P < 0.0001. (**H**) Western blot analysis of PD-L1 protein levels in 786-O and H1299 cells treated with butyrate (4 mM) or actinomycin D (AD, 5 μg/mL) for 24 h. (**I**) qRT-PCR analysis of *PD-L1* levels in 786-O and H1299 cells treated with butyrate (4 mM) or actinomycin D (AD, 5 μg/mL) for 24 h. Values are means ± SD from n = 3 independent experiments. Statistical differences were determined by ordinary one-way ANOVA. ns, nonsignificant, *P < 0.05, **P < 0.01, ****P < 0.0001.

To validate whether butyrate-induced PD-L1 upregulation is a broad phenomenon across different tumor types, we treated a panel of human and murine tumor cell lines with butyrate, including human renal carcinoma cells (786-O), human non-small cell lung cancer cells (H1299), human breast cancer cells (MDA-MB-231 and BT-549), murine breast cancer cells (4T1), human cervical cancer cells (HeLa), human melanoma cells (A375), human pancreatic cancer cells (SW1990), human hepatocellular carcinoma cells (HCCLM3 and MHCC97H), murine hepatoma cells (Hepa 1-6), and murine colorectal cancer cells (CT26). We observed a consistent and significant upregulation of PD-L1 protein levels across these tumor cell lines (Fig. 2A). This induction followed both time- (Fig. 2B) and dose-dependent (Fig. 2C) patterns, demonstrating a dynamic and regulated response. Flow cytometry analysis further confirmed that butyrate specifically enhanced cell surface PD-L1 expression (Fig. 2D), reinforcing its potential role in modulating tumor-immune interactions. Together, these results conclusively demonstrate that butyrate is a potent inducer of PD-L1 expression in tumor cells.

### Butyrate upregulates PD-L1 expression through transcriptional activation

To elucidate the mechanism underlying butyrate-induced PD-L1 upregulation, we investigated its effect on *PD-L1* transcription. qRT-PCR analysis revealed that butyrate treatment significantly increased *PD-L1* mRNA levels across multiple tumor cell lines (Fig. 2E). This enhancement exhibited both time- (Fig. 2F) and concentration-dependent (Fig. 2G) profiles. To substantiate these findings, we employed actinomycin D, a potent inhibitor of transcription, in subsequent experiments. Actinomycin D treatment effectively abolished the butyrate-mediated increase in both PD-L1 protein expression (Fig. 2H) and mRNA levels (Fig. 2I). Overall, these results demonstrate that butyrate upregulates PD-L1 protein expression by activating its transcription.

### Butyrate upregulates PD-L1 through HDAC inhibition-dependent epigenetic remodeling

**Figure 3:**
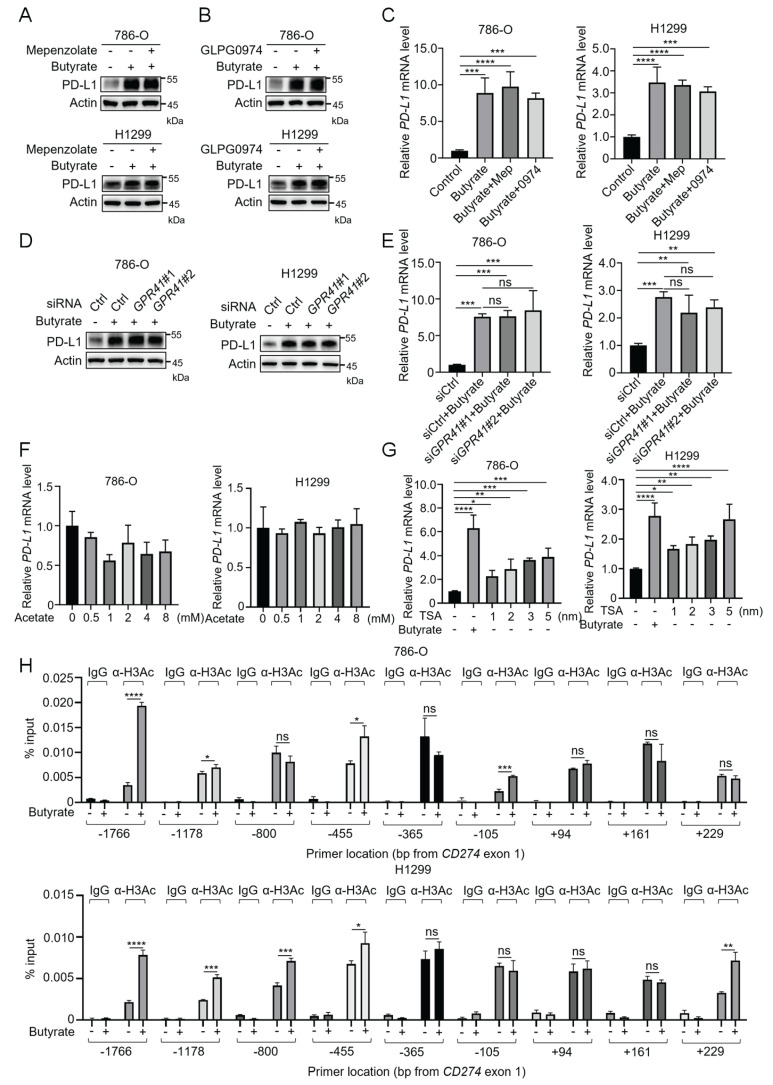
** Butyrate upregulates PD-L1 through HDAC inhibition-dependent epigenetic remodeling.** (**A**) Western blot analysis of PD-L1 protein levels in 786-O and H1299 cells treated with butyrate (4 mM, 48 h) or mepenzolate bromide (Mep, 100 μg/mL, 54 h). (**B**) Western blot analysis of PD-L1 protein levels in 786-O and H1299 cells treated with butyrate (4 mM, 48 h) or GLPG0974 (0974, 0.1 μM, 54 h). (**C**) qRT-PCR analysis of *PD-L1* levels in 786-O and H1299 cells treated with butyrate (4 mM, 48 h), mepenzolate bromide (Mep, 100 μg/mL, 54 h), or GLPG0974 (0974, 0.1 μM, 54 h). Values are means ± SD from n = 3 independent experiments. Statistical differences were determined by ordinary one-way ANOVA. ***P < 0.001, ****P < 0.0001. (**D**) Western blot analysis of PD-L1 expression in 786-O and H1299 cells transfected with control or *GPR41*-targeting siRNAs for 24 h before treatment with butyrate (4 mM) for an additional 24 h. (**E**) qRT-PCR analysis of *PD-L1* mRNA expression in 786-O and H1299 cells transfected with control or *GPR41*-targeting siRNAs for 24 h before treatment with butyrate (4 mM) for an additional 24 h. Values are means ± SD from n = 3 independent experiments. Statistical differences were determined by ordinary one-way ANOVA. ns, nonsignificant, **P < 0.01, ***P < 0.001. (**F**) qRT-PCR analysis of *PD-L1* levels in 786-O and H1299 cells treated with different concentrations of acetate as indicated for 48 h. (**G**) qRT-PCR analysis of *PD-L1* levels in 786-O and H1299 cells treated with butyrate (4 mM) or TSA for 24 h. Values are means ± SD from n = 3 independent experiments. Statistical differences were determined by ordinary one-way ANOVA. *P < 0.05, **P < 0.01, ***P < 0.001, ****P < 0.0001. (**H**) ChIP assay was used to detect histone H3Ac levels in the promoter region of *CD274* in 786-O and H1299 cells treated with butyrate (4 mM) for 12 h. IgG serves as a negative control. Values are means ± SD from n = 3 independent experiments. Statistical differences were determined by Student's *t* test. ns, nonsignificant, *P < 0.05, **P < 0.01, ***P < 0.001, ****P < 0.0001.

Butyrate exerts biological effects partly through signaling via GPCRs, including GPR41, GPR43, and GPR109A^21^. To investigate whether these GPCRs mediate butyrate-induced PD-L1 upregulation, we employed specific inhibitors: mepenzolate bromide (GPR109A inhibitor) and GLPG0974 (GPR43 inhibitor). Treatment with these two inhibitors failed to attenuate butyrate-induced increases in PD-L1 protein expression (Fig. 3A and 3B) or mRNA levels (Fig. 3C). Similarly, siRNA-mediated knockdown of GPR41 (Fig. S2A) did not affect butyrate's ability to upregulate PD-L1 protein (Fig. 3D) or transcription (Fig. 3E) levels. These results collectively demonstrate that butyrate regulates PD-L1 independently of GPCR signaling.

An alternative mechanism by which butyrate functions is through inhibition of HDAC activity, thereby modulating gene expression. Treating 786-O and H1299 cells with butyrate significantly elevated global histone H3Ac levels in a time-dependent manner (Fig. S2B), which is consistent with reported research results^8^. At the same time, we evaluated acetate, a structurally related SCFA lacking HDAC inhibitory activity. The results showed that acetate treatment did not affect the protein (Fig. S2C) or mRNA (Fig. 3F) levels of PD-L1. This provides indirect evidence that butyrate's regulation of PD-L1 is attributable to its HDAC inhibitory function. Supporting this, the classic HDAC inhibitor trichostatin A (TSA), like butyrate, significantly upregulated both PD-L1 protein (Fig. S2D) and mRNA expression (Fig. 3G). Furthermore, chromatin immunoprecipitation (ChIP) assays confirmed that butyrate treatment specifically enhanced histone H3Ac enrichment within the *PD-L1* promoter region (Fig. 3H). Taken together, these data establish that butyrate acts as an HDAC inhibitor to enhance histone H3Ac at the *PD-L1* promoter, thereby driving its transcriptional upregulation.

### Butyrate promotes PD-L1 expression partially via TGM2

**Figure 4:**
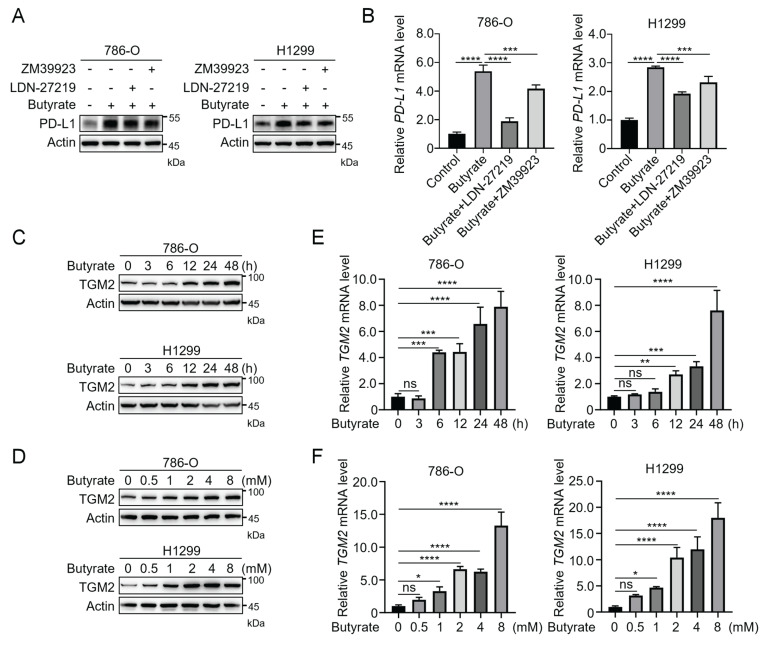
** Butyrate promotes PD-L1 expression partially via TGM2.** (**A**) Western blot analysis of PD-L1 protein levels in 786-O and H1299 cells treated with butyrate (4 mM, 48 h), ZM39923 (20 nM, 54 h), or LDN-27219 (5 μM, 54 h). (**B**) qRT-PCR analysis of *PD-L1* levels in 786-O and H1299 cells treated with butyrate (4 mM, 48 h), ZM39923 (20 nM, 54 h), or LDN-27219 (5 μM, 54 h). Values are means ± SD from n = 3 independent experiments. Statistical differences were determined by ordinary one-way ANOVA. ***P < 0.001, ****P < 0.0001. (**C**) Western blot analysis of TGM2 protein levels in 786-O and H1299 cells treated with 4 mM butyrate for different times as indicated. (**D**) Western blot analysis of TGM2 protein levels in 786-O and H1299 cells treated with different concentrations of butyrate as indicated for 48 h. (**E**) qRT-PCR analysis of *TGM2* levels in 786-O and H1299 cells treated with 4 mM butyrate for different times as indicated. Values are means ± SD from n = 3 independent experiments. Statistical differences were determined by ordinary one-way ANOVA. ns, nonsignificant, **P < 0.01, ***P < 0.001, ****P < 0.0001. (**F**) qRT-PCR analysis of *TGM2* levels in 786-O and H1299 cells treated with different concentrations of butyrate as indicated for 48 h. Values are means ± SD from n = 3 independent experiments. Statistical differences were determined by ordinary one-way ANOVA. ns, nonsignificant, *P < 0.05, ****P < 0.0001.

While our ChIP experiments established that butyrate upregulates *PD-L1* via enhanced histone H3Ac at its promoter, we investigated potential additional mechanisms regulating PD-L1 expression. We noticed from the RNA-seq data that TGM2 expression was markedly induced by butyrate (Fig. 1C). TGM2, a multifunctional enzyme implicated in extracellular matrix remodeling, apoptosis, and signal transduction^22^, ^23^, also exhibited a strong positive correlation with PD-L1 across multiple cancer types in the TCGA database (Fig. S3). To functionally assess this association, we inhibited TGM2 activity using ZM39923 and LDN-27219. WB analysis demonstrated that TGM2 inhibition partially attenuated, but did not fully abrogate, the butyrate-induced increase in PD-L1 protein levels (Fig. 4A). Similarly, TGM2 inhibition partially reversed the upregulation of *PD-L1* mRNA by butyrate, though transcript levels remained elevated compared to controls (Fig. 4B). Consistent with the RNA-seq data, butyrate treatment robustly increased both TGM2 protein (Fig. 4C and 4D) and mRNA (Fig. 4E and 4F) expression in time- and concentration-dependent manners. It is noteworthy that we also employed iodoacetamide (IA), a nonspecific irreversible inhibitor of TGM2. In line with the preceding findings, IA partially suppressed the butyrate-induced upregulation of PD-L1 expression (Fig. S4A and S4B). Furthermore, the upregulation of TGM2 by butyrate was observed broadly, both at the protein (Fig. S4C) and transcriptional levels (Fig. S4D). Together, these results identify TGM2 as a functional mediator in the upregulation of PD-L1 driven by butyrate.

### TGM2 upregulates *PD-L1* transcription by promoting histone H3K4me3 modification

**Figure 5:**
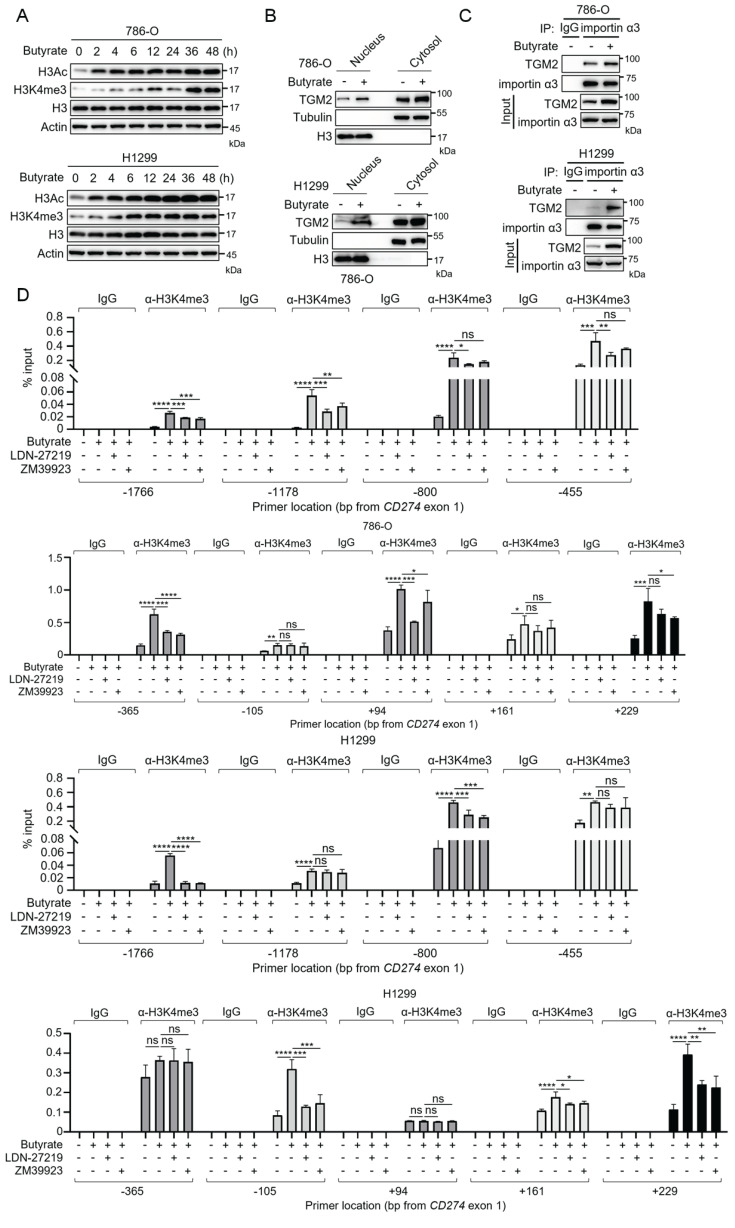
** TGM2 upregulates PD-L1 transcription by promoting histone H3K4me3 modification.** (**A**) Western blot analysis of histone H3, H3Ac, and H3K4me3 protein levels in 786-O and H1299 cells treated with 4 mM butyrate for different times as indicated. (**B**) Western blot analysis of the cytosolic and nuclear localization of TGM2 in 786-O and H1299 cells treated with butyrate (4 mM, 48 h). (**C**) Assay of TGM2 interaction with importin α3. 786-O and H1299 cells were treated with 4 mM butyrate for 48 h. IgG serves as a negative control. (**D**) ChIP assay was used to detect histone H3K4me3 levels in the promoter region of *CD274* in 786-O and H1299 cells treated with butyrate (4 mM, 12 h), ZM39923 (20 nM, 18 h), or LDN-27219 (5 μM, 18 h). IgG serves as a negative control. Values are means ± SD from n = 3 independent experiments. Statistical differences were determined by ordinary one-way ANOVA. ns, nonsignificant, *P < 0.05, **P < 0.01, ***P < 0.001, ****P < 0.0001.

Previous studies have identified TGM2-mediated serotonylation of glutamine at position 5 on histone H3 (H3Q5ser) as the first discovered endogenous monoaminyl post-translational modification^24^. This modification frequently co-localizes with H3K4me3, as it facilitates TFIID binding to H3K4me3-marked regions^24^, ^25^. To investigate whether TGM2 potentiates butyrate-induced *PD-L1* transcription through this epigenetic mechanism, we first observed that butyrate treatment time-dependently increased global H3K4me3 modification levels (Fig. 5A). Given the nuclear localization of histones, TGM2 requires nuclear entry to exert its histone-modifying function. Subcellular fractionation followed by WB analysis revealed that butyrate significantly promoted TGM2 nuclear translocation (Fig. 5B). As importin α3 has been reported to mediate TGM2 nuclear import^26^, we performed co-immunoprecipitation (Co-IP) assays and confirmed that butyrate enhances the interaction between importin α3 and TGM2 (Fig. 5C). To functionally link TGM2 activity to H3K4me3 enrichment at the *PD-L1* locus, we conducted ChIP assays using an H3K4me3-specific antibody. Butyrate treatment robustly increased H3K4me3 enrichment in the *PD-L1* promoter region, while pharmacological inhibition of TGM2 partially reduced this enrichment (Fig. 5D). This partial reduction aligns with our previous observations of attenuated PD-L1 upregulation upon TGM2 inhibition. Collectively, these findings suggest that butyrate induces nuclear import of TGM2 via importin α3, where TGM2 is proposed to promote H3Q5 serotonylation, thereby facilitating H3K4me3 deposition, thereby amplifying *PD-L1* transcription and elevating PD-L1 protein expression.

### Butyrate enhances anti-PD-L1 immunotherapy for colorectal cancer *in vivo*

While monoclonal antibodies targeting immune checkpoints represent landmark advances in cancer therapy^16^, expanding the population of patients benefiting from anti-PD-1/PD-L1 blockade remains a major clinical challenge. This is particularly relevant for patients exhibiting low response rates, which are strongly associated with low tumor PD-L1 expression^27^. Moreover, accumulating evidence establishes PD-L1 as a critical biomarker for predicting immunotherapy efficacy^28^, ^29^. Therefore, elucidating the molecular mechanisms governing PD-L1 expression and/or stability in tumor cells is essential for improving clinical responses to immunotherapy. Our findings demonstrating butyrate-mediated upregulation of PD-L1 in tumor cells suggest its potential to modulate immunotherapy outcomes.

**Figure 6:**
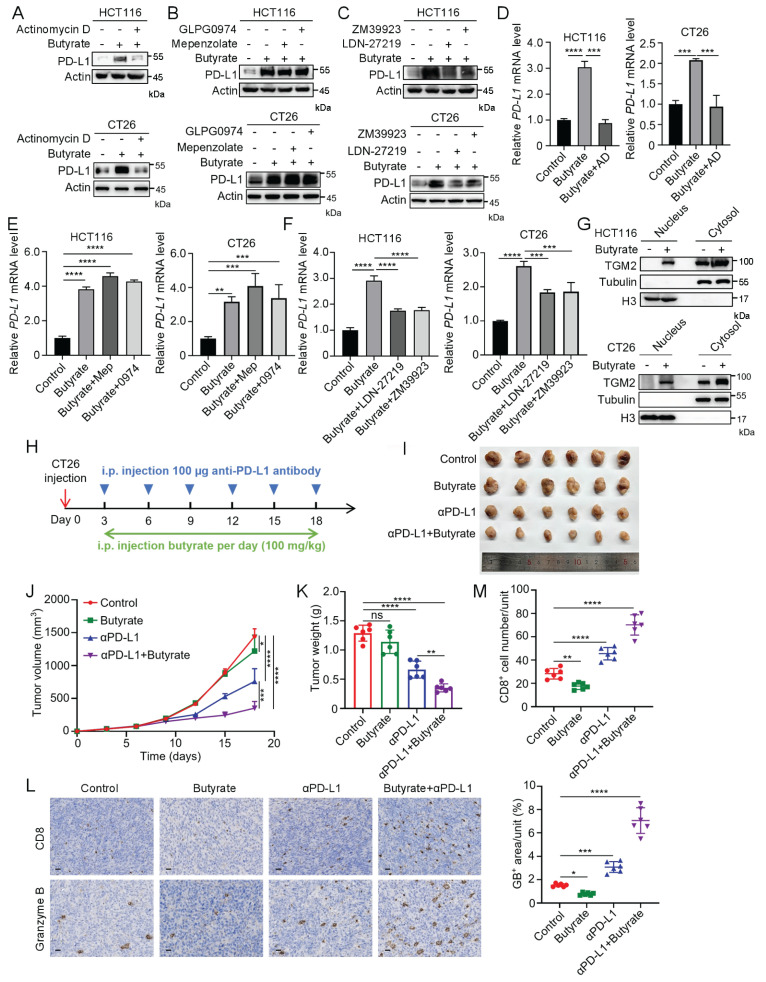
** Butyrate enhances anti-PD-L1 immunotherapy for colorectal cancer *in vivo*.** (**A**) Western blot analysis of PD-L1 protein levels in HCT116 and CT26 cells treated with butyrate (4 mM) or actinomycin D (AD, 5 μg/mL) for 24 h. (**B**) Western blot analysis of PD-L1 protein levels in HCT116 and CT26 cells treated with butyrate (4 mM, 48 h), mepenzolate (Mep, 100 μg/mL, 54 h), or GLPG0974 (0974, 0.1 μM, 54 h). (**C**) Western blot analysis of PD-L1 protein levels in HCT116 and CT26 cells treated with butyrate (4 mM, 48 h), ZM39923 (20 nM, 54 h), or LDN-27219 (5 μM, 54 h). (**D**) qRT-PCR analysis of *PD-L1* levels in HCT116 and CT26 cells treated with butyrate (4 mM) or actinomycin D (AD, 5 μg/mL) for 24 h. Values are means ± SD from n = 3 independent experiments. Statistical differences were determined by ordinary one-way ANOVA. ***P < 0.001, ****P < 0.0001. (**E**) qRT-PCR analysis of *PD-L1* levels in HCT116 and CT26 cells treated with butyrate (4 mM, 48 h), mepenzolate (Mep, 100 μg/mL, 54 h), or GLPG0974 (0974, 0.1 μM, 54 h). Values are means ± SD from n = 3 independent experiments. Statistical differences were determined by ordinary one-way ANOVA. **P < 0.01, ***P < 0.001, ****P < 0.0001. (**F**) qRT-PCR analysis of *PD-L1* levels in HCT116 and CT26 cells treated with butyrate (4 mM, 48 h), ZM39923 (20 nM, 54 h), or LDN-27219 (5 μM, 54 h). Values are means ± SD from n = 3 independent experiments. Statistical differences were determined by ordinary one-way ANOVA. ***P < 0.001, ****P < 0.0001. (**G**) Western blot analysis of the cytosolic and nuclear localization of TGM2 in HCT116 and CT26 cells treated with butyrate (4 mM, 48 h). (**H**) Schematic representation of the animal experiment process. (**I**) CT26 cells in BALB/c mice treated with butyrate or anti-PD-L1 antibodies. Tumors were resected from each group of mice that received different treatments as indicated. n = 6 mice per group. (**J**) Tumor growth of CT26 cells in BALB/c mice was determined. Statistical differences were determined by ordinary one-way ANOVA. *P < 0.05, ***P < 0.001, ****P < 0.0001. (**K**) The weight of tumors resected from each group of mice that received different treatments as indicated was analyzed. Data represent mean ± SD, n = 6 mice per group. Statistical differences were determined by ordinary one-way ANOVA. ns, nonsignificant, **P < 0.01, ****P < 0.0001. (**L**) Immunohistochemistry showing CD8 and granzyme B levels in the CT26 tumor tissues as indicated (Scale bars, 20 μm). (**M**) Quantifications of images in (L). Data represent mean ± SD from six independent samples of each group. Statistical differences were determined by ordinary one-way ANOVA. *P < 0.05, **P < 0.01, ***P < 0.001, ****P < 0.0001.

We first confirmed butyrate-induced PD-L1 upregulation in the human colon cancer cell line HCT116 and the mouse colon cancer cell line CT26 (Fig. 6A-G). Subsequently, we established a subcutaneous CT26 tumor model in BALB/c mice (Fig. 6H) to evaluate the immunotherapeutic effect of butyrate. Monotherapy with anti-PD-L1 antibodies significantly inhibited tumor growth. Strikingly, the combination of butyrate and anti-PD-L1 antibodies resulted in markedly enhanced tumor suppression compared to either treatment alone (Fig. 6I-K). Immunohistochemical (IHC) analysis of tumor tissues further revealed that the combination therapy significantly increased tumor-infiltrating CD8^+^ T cell numbers and granzyme B levels (Fig. 6L and 6M), indicative of enhanced cytotoxic T cell activity. Consistently, Western blot analysis of tumor tissues from this model demonstrated that butyrate treatment increased PD-L1 protein levels* in vivo* (Fig. S5A), supporting the translational relevance of our *in vitro* findings. In summary, these* in vivo* results demonstrate that butyrate potentiates the efficacy of anti-PD-L1 immunotherapy. This supports its potential as a sensitizing agent for PD-L1 blockade, offering a promising strategy to improve outcomes for cancer patients whose tumors exhibit PD-L1-negative or low expression.

### High-fiber diets enhance immunotherapy for AOM-DSS-induced CRC

**Figure 7:**
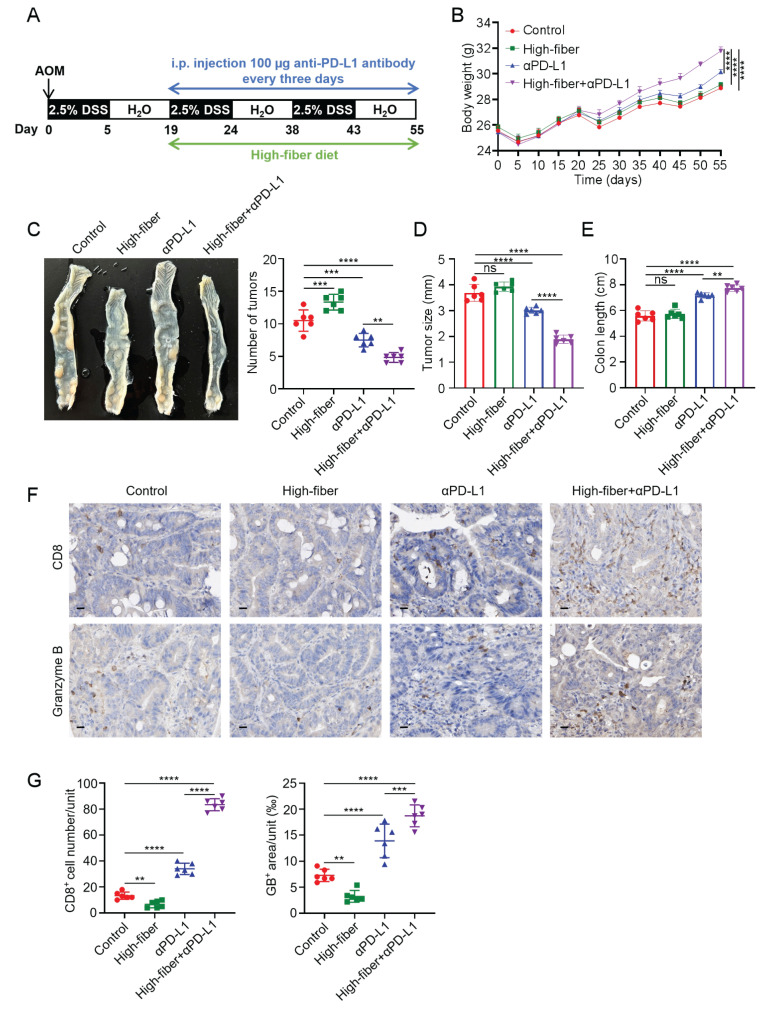
** High-fiber diets enhance immunotherapy for AOM-DSS-induced CRC.** (**A**) Schematic representation of the animal experiment process. (**B**) Body weight changes of mice in each group. Data represent mean ± SD, n = 6 mice per group. Statistical differences were determined by ordinary one-way ANOVA. ****P <0.0001. (**C**) Representative images of mouse colons are shown, and tumor numbers are quantified. Data represent mean ± SD, n = 6 mice per group. Statistical differences were determined by ordinary one-way ANOVA. **P < 0.01, ***P < 0.001, ****P <0.0001. (**D**) The diameters of all tumors per mouse were measured, averaged, and the tumor sizes of the four groups were statistically analyzed. Data represent mean ± SD, n = 6 mice per group. Statistical differences were determined by ordinary one-way ANOVA. ns, nonsignificant, ****P <0.0001. (**E**) Colon length of mice in each group. Data represent mean ± SD, n = 6 mice per group. Statistical differences were determined by ordinary one-way ANOVA. ns, nonsignificant, **P < 0.01, ****P <0.0001. (**F**) Immunohistochemistry showing CD8 and granzyme B levels in the tumor tissues as indicated (Scale bars, 20 μm). (**G**) Quantifications of images in (F). Data represent mean ± SD from six independent samples of each group. Statistical differences were determined by ordinary one-way ANOVA. **P < 0.01, ***P < 0.001, ****P < 0.0001.

Given that gut microbiota ferment dietary fiber to produce butyrate, we investigated whether high-fiber diets could similarly enhance tumor immunotherapy efficacy. We established an AOM-DSS-induced CRC model in mice (Fig. 7A) and administered customized diets formulated with isocaloric composition (Supplementary Table 1). The high-fiber diet contained inulin, a substrate known to yield butyrate upon intestinal fermentation^30^. Strikingly, compared with the model group, the combination of a high-fiber diet and anti-PD-L1 antibody significantly restored mouse body weight (Fig. 7B), while markedly reducing both the number and size of colonic tumors (Fig. 7C and 7D). Furthermore, this combined treatment significantly preserved colon length (Fig. 7E), a critical indicator of AOM-DSS-induced CRC progression. Consistent with these findings, IHC analysis of tumor tissues revealed markedly increased CD8^+^ T cell infiltration and elevated granzyme B levels in the combination therapy group (Fig. 7F and 7G). Mechanistically, Western blot analysis of tumor tissues showed that both PD-L1 and TGM2 were upregulated in the high-fiber group (Fig. S5B). Furthermore, increased levels of H3Ac and H3K4me3 were observed (Fig. S5C), indicating that epigenetic modulation contributes to PD-L1 regulation *in vivo*. These findings demonstrate that a high-fiber diet, a cost-effective and clinically implementable strategy, effectively synergizes with anti-PD-L1 immunotherapy, mirroring the effects of butyrate. Further research is warranted to explore the feasibility of dietary modulation for enhancing immunotherapy responses in humans.

## Discussion

Evading immune surveillance is one of the fundamental characteristics of tumors. Tumors frequently activate the PD-L1/PD-1 pathway within the tumor microenvironment (TME) to subvert immune responses^30^. Consequently, monoclonal antibodies targeting PD-L1/PD-1 have demonstrated significant clinical efficacy across multiple malignancies. However, a significant proportion of patients exhibit limited therapeutic response. Elevated tumor PD-L1 expression is a well-established biomarker predictive of enhanced sensitivity to PD-1/PD-L1 blockade^31^, ^32^. Corroborating this, clinical trials evaluating the anti-PD-L1 antibody MPDL3280A revealed that disease progression frequently correlated with absent PD-L1 expression in tumor biopsies^33^. Thus, PD-L1 negativity or low expression represents an important determinant of resistance to PD-L1/PD-1-targeted therapies.

Our findings demonstrate that butyrate significantly upregulates PD-L1 expression across various tumor cells. Consequently, combining butyrate or high-fiber diets that boost microbial butyrate production with anti-PD-L1 blockade profoundly suppresses CRC progression in murine models. These findings suggest that butyrate-mediated PD-L1 upregulation may enhance tumor sensitivity to PD-L1 blockade, particularly in tumors with relatively low baseline PD-L1 expression, thereby highlighting the importance of patient stratification based on tumor immune contexture. However, caution is warranted; without concurrent checkpoint blockade, butyrate-induced PD-L1 upregulation risks promoting tumor immune evasion.

Mechanistically, butyrate and anti-PD-L1 therapies may exert distinct effects on CD8^+^ T cell dynamics and function within the tumor microenvironment. Butyrate-induced PD-L1 upregulation could suppress the cytotoxic effector function of infiltrating CD8^+^ T cells through PD-1 engagement within the tumor microenvironment. In this context, anti-PD-L1 antibodies may primarily restore T cell activity rather than simply increasing T cell numbers. Therefore, the enhanced anti-tumor efficacy observed with combination therapy may result from the restoration of CD8^+^ T cell function together with amplification of anti-tumor immune responses. This interpretation is also supported by the increased granzyme B expression observed in the combination treatment group. Notably, although butyrate-induced PD-L1 upregulation may suppress T cell-mediated cytotoxicity, butyrate monotherapy still exhibited partial anti-tumor activity *in vivo*. This may reflect the pleiotropic effects of butyrate within the tumor microenvironment, as butyrate can regulate multiple immune and tumor-associated pathways beyond the PD-L1 axis. Therefore, the overall effect of butyrate *in vivo* likely depends on the balance among these context-dependent mechanisms.

Two main limitations must be acknowledged. First, our murine models (including the syngeneic CT26/BALB/c model and the AOM-DSS-induced CRC model) may not fully recapitulate the complexity of the human tumor immune microenvironment or human-specific immune checkpoint interactions. Therefore, further validation in humanized mouse models and clinical samples will be important to strengthen the translational relevance of our findings. Second, as a broad HDAC inhibitor, butyrate exerts pleiotropic epigenetic effects; thus, its immunomodulatory role likely extends beyond the PD-L1 axis.

Histone post-translational modifications (PTMs) play a pivotal role in regulating DNA-templated processes, including gene transcription^34^, ^35^. Among the most well-characterized epigenetic markers, H3Ac and H3K4me3 are evolutionarily conserved PTMs linked to transcriptional activation. These modifications are highly enriched at promoter regions and transcription start sites, where they facilitate chromatin remodeling and transcriptional machinery recruitment^36^, ^37^. Recent studies have identified a novel histone post-translational modification involving TGM2-mediated conjugation of 5-hydroxytryptamine (5-HT) to H3 glutamine-5 (Q5) within H3K4me3-marked nucleosomes, forming the H3K4me3Q5ser modification that activates transcription^24^. Mechanistically, H3Q5ser adjacent to H3K4me3 functions cooperatively by stabilizing H3K4me3 by reducing its turnover rate, and enhancing recognition by downstream reader proteins. This dual-action mechanism enables precise regulation of critical gene expression programs^25^.

Here, we propose a model wherein TGM2 catalyzes H3Q5ser modification, thereby potentiating H3K4me3 enrichment at the *PD-L1* promoter to drive transcription. However, in this hypothetical process, which requires 5-HT as the substrate for histone modification by TGM2, it remains unclear whether 5-HT is endogenously produced by cells or if the trace amounts present in the culture medium might be sufficient to mediate such histone modifications. Interestingly, emerging evidence indicates that L-5-hydroxytryptophan (L-5-HTP, precursor of 5-HT) suppresses interferon-gamma (IFN-γ)-induced PD-L1 expression^38^, which appears to be inconsistent with our findings. These discrepancies may reflect context-dependent differences in tumor microenvironment, particularly variations in IFN-γ signaling. However, we hypothesize that butyrate selectively enhances immunotherapy efficacy in patients with low baseline PD-L1 expression, particularly those with an immune “cold” tumor microenvironment characterized by reduced IFN-γ signaling. Collectively, these observations highlight the dual role of butyrate in tumor immunity. Moreover, although our findings are supported by multiple experimental approaches and tumor cell models, further validation in additional preclinical systems and clinical samples will be necessary to confirm the reproducibility and generalizability of these observations. On one hand, butyrate-induced PD-L1 upregulation may sensitize tumors to immune checkpoint blockade; on the other hand, in the absence of such therapy, increased PD-L1 expression may facilitate tumor immune evasion by suppressing anti-tumor immune responses. Therefore, the therapeutic impact of butyrate is highly context-dependent, and personalized treatment strategies will be required for different patient populations.

In summary, our study proposes a potential strategy to enhance PD-L1 blockade efficacy in tumors with relatively low PD-L1 expression. Mechanistically, butyrate orchestrates dual histone modifications at the *PD-L1* promoter: (1) HDAC inhibition elevates histone H3Ac, and (2) TGM2 upregulation promotes histone H3Q5ser, which synergistically enhances histone H3K4me3 deposition to amplify transcription. While the precise pathway driving butyrate-induced TGM2 transcription requires further elucidation, HDAC inhibition may play a role in this process. Importantly, both butyrate and high-fiber diets significantly enhanced anti-PD-L1 efficacy in CRC models. Butyrate supplementation or high-fiber dietary intervention may represent a potential adjunctive strategy to enhance anti-PD-L1 immunotherapy efficacy, although further preclinical and clinical validation will be required. Moreover, careful patient selection may be important to maximize therapeutic benefit while minimizing the potential risk of tumor immune escape.

## Materials and Methods

### Cell culture

786-O, MDA-MB-231, A375, SW1990, HCCLM3, MHCC97H, HeLa, and Hepa 1-6 cells were cultured in DMEM medium containing FBS (10%) and penicillin/streptomycin (P/S, 1%). H1299, BT-549, 4T1, and CT26 were cultured in DMEM medium containing FBS (10%) and P/S (1%). All cells were cultured in a 37℃ constant temperature incubator containing 5% CO_2_. The compounds used in this study included sodium butyrate (Sigma-Aldrich, V900464, reagent grade, purity 99%), actinomycin D (MCE, HY-17559, purity 98.12%), trichostatin A (MCE, HY-15144, purity 99.85%), mepenzolate bromide (MCE, HY-17585, purity 99.90%), sodium acetate (Sigma-Aldrich, 241245-5G, ACS reagent, purity ≥99.0%), GLPG0974 (MCE, HY-12940, purity 98.12%), ZM39923 hydrochloride (MCE, HY-12589, purity 99.84%), LDN-27219 (MCE, HY-16693, purity 99.76%), and 2-Iodoacetamide (MCE, HY-34477, purity 99.62%).

### siRNA transfection

jetPRIME® Transfection Reagent (Polyplus, 101000046) was used for siRNA transfection. All operations were performed according to the manufacturer's instructions. Cells were transiently transfected with siRNAs (siRNAs against Control, *GPR41*#1, and *GPR41*#2 were commercially synthesized; Shanghai GenePharma), and incubated in cell culture medium for 6 hours. siControl: sense, 5′-UUCUCCGAACGUGUCACGU-3′, antisense, 5′-ACGUGACACGUUCGGAGAA-3′; si*GPR41*#1: sense, 5′-CAGUUCUGCCGGAUAAAUA-3′, antisense, 5′-UAUUUAUCCGGCAGAACUG-3′; si*GPR41*#2: sense, 5′-CAACAGUGAGUGACGUCAU-3′, antisense, 5′-AUGACGUCACUCACUGUUG-3′.

### Cell gene knockout

*PD-L1* was knocked out using the CRISPR/Cas9 system. Three plasmids, LentiCRISPRv2, psPAX2, and pMD2, were co-transfected into HEK 293T cells in a 4:3:1 ratio, and cell supernatants were collected at 48 and 72 hours. Tumor cells were cultured in a medium containing polybrene (10 μg/mL) and lentivirus for 48 hours, and then selected with puromycin. The guide RNA sequence targeting *PD-L1* was designed following previous work^39^.

### Western blot and coimmunoprecipitation

The steps for preparing cell lysate and Western blot (WB) refer to our previous work^40^. The antibody was incubated with Protein G Sepharose (Cytiva) at 4℃ for 3 hours, and centrifuged (2000 rpm, 5 min, 4℃) to remove the supernatant. The cell lysates were incubated with the precipitate at 4℃ overnight, centrifuge (10000 rpm, 5 min, 4℃) to remove the supernatant, and clean the precipitate 6 times. Lysis buffer was added, containing loading buffer to the precipitation and boil the sample at 100℃ for 10 min. After centrifugation (12000 rpm, 5 min, 4℃), the supernatant was gently aspirated to obtain the sample for WB. The antibodies used in this study include PD-L1 (CST, 51296; Invitrogen, PA5-28115), mouse-PD-L1 (Abcam, ab213480), TGM2 (Proteintech, 15100-1-AP), importin α3 (Proteintech, 12463-1-AP), H3Ac (Sigma-Aldrich, 17-615), tri-methyl-histone H3 (Lys4) (CST, C42D8), H3 (HuaBio, M1309-1), Tubulin (Proteintech, 10068-1-AP), Actin (HuaBio, EW21002), PD-L2 (Abcam, ab288298), B7-H3 (CST, 14058T), and B7-H4 (CST, D1M8I).

### Quantitative real-time PCR

RNA purification kit (EZBioscience) and Reverse Transcription Master Mix (EZBioscience) were used to extract total RNA from cells and perform reverse transcription reactions, respectively. qRT-PCR experiments were performed using Applied Biosystems 7300 Sequence Detection System (Applied Biosystems). The following primers were used *hβ-actin*: forward, 5′-GGCATAGAGGTCTTTACGGATGTC-3′, reverse, 5′-TATTGGCAACGAGCGGTTCC-3′; *mβ-actin*: forward, 5′- GGCTGTATTCCCCTCCATCG-3′, reverse, 5′-CCAGTTGGTAACAATGCCATGT-3′; *hPD-L1*: forward, 5′-TGGCATTTGCTGAACGCATTT-3′, reverse, 5′-TGCAGCCAGGTCTAATTGTTTT-3′; *mPD-L1*: forward, 5′- GCTCCAAAGGACTTGTACGTG-3′, reverse, 5′- TGATCTGAAGGGCAGCATTTC-3′; *hTGM2*: forward, 5′-GAGGAGCTGGTCTTAGAGAGG-3′, reverse, 5′-CGGTCACGACACTGAAGGTG-3′; *mTGM2*: forward, 5′-GACAATGTGGAGGAGGGATCT-3′, reverse, 5′-CTCTAGGCTGAGACGGTACAG-3′; *hGPR41*: forward, 5′-TTCACCACCATCTATCTCACCG-3′, reverse, 5′-GGAACTCCAGGTAGCAGGTC-3′.

### Separation of nuclear and cytoplasmic proteins

The nucleus and cytoplasm of cells were separated using the nuclear protein and cytoplasmic protein extraction kit (Beyotime, P0027), and prepare whole cell lysate for WB. All operations were carried out according to the instructions.

### Chromatin immunoprecipitation (ChIP)

All operational steps of this experiment were carried out according to the SimpleChIP Plus Enzymatic Chromatin IP Kit (CST). The obtained DNA was used for qRT-PCR. The following primers are used for qRT-PCR: *CD274* promoter-1 (-1766, bp from *CD274* exon 1): forward, 5′-GGCAAATTCCGTTTGCCTCA-3′, reverse, 5′-TCCTCCTAGATGGCCTGGAT-3′; *CD274* promoter-2 (-1178): forward, 5′-GCTGGGCCCAAACCCTATT-3′, reverse, 5′-TTTGGCAGGAGCATGGAGTT-3′; *CD274* promoter-3 (-800): forward, 5′-CTAGAAGTTCAGCGCGGGAT-3′, reverse, 5′-GGCCCAAGATGACAGACGAT-3′; *CD274* promoter-4 (-455): forward, 5′-ATGGGTCTGCTGCTGACTTT-3′, reverse, 5′-GGCGTCCCCCTTTCTGATAA-3′; *CD274* promoter-5 (-365): forward, 5′-GGGGGACGCCTTTCTGATAA-3′, reverse, 5′-AAGCCAACATCTGAACGCAC-3′; *CD274* promoter-6 (-105): forward, 5′-ACTGAAAGCTTCCGCCGATT-3′, reverse, 5′-CCCAAGGCAGCAAATCCAGT-3′; *CD274* promoter-7 (+94): forward, 5′-GTAGGGAGCGTTGTTCCTCC-3′, reverse, 5′-GTGTAGAGACCCTCCGTCCT-3′; *CD274* promoter-8 (+161): forward, 5′-AGGACGGAGGGTCTCTACAC-3′, reverse, 5′-ATTGGCTCTACTGCCCCCTA-3′.

### RNA-seq and data analysis

786-O cells were pretreated with butyrate (4 mM) for 48 h. Total RNA was extracted from 786-O cells for preparation of the cDNA library. RNA-seq analysis was conducted by GENEWIZ (Suzhou, China).

### Flow cytometry

Tumor cells were incubated with APC-conjugated anti-human CD274 antibody (BioLegend) in the dark for 30 minutes on ice, and then the cells were washed twice with PBS. Finally, stained cells were analyzed using BD FACSCelesta, and the data were processed with FlowJo.

### Animal experiments

The animal experiments in this study conform to the ethical review approved by the Department of Experimental Animal Science of Fudan University. Female BALB/c mice (6- to 8-week-old) were purchased from GemPharmatech Laboratory Animals. Mice live in a clean environment at 22-23℃, with a 12-hour light/dark cycle. They can freely feed and drink water. CT26 cells (5 × 10^5^) were subcutaneously injected into the right flank of mice and randomly divide them into four groups. Butyrate (100 mg/kg, once a day) and anti-PD-L1 antibody (100 μg, once every two days, Bioxcell, BE0101) were administered intraperitoneally. The length and width of tumor tissue were measured every three days using a vernier caliper, and the tumor volume was calculated according to the formula: length/2 × width^2^.

8-week-old male mice were purchased from the Hangzhou Qizhen Laboratory Animal Co., Ltd. To construct the AOM-DSS-induced CRC model, azoxymethane (AOM, 10 mg/kg, MCE, HY-111375) was intraperitoneally injected on the first day, fed with 2.5% DSS (MP Biomedicals, U1122304862-2) drinking water for five days and regular drinking water for 14 days, for three cycles. At the beginning of the second cycle, the mouse diet was switched to a high-fiber diet or an equivalent calorie control group, and 100 μg of anti-PD-L1 antibody was intraperitoneally injected every three days. On the 55th day, mice were euthanized, and colon tissue was taken for measurement and photography, followed by immunohistochemistry (IHC) analysis. IHC analysis has been described in previously published work^41^, ^42^.

### T-cell-mediated tumor cell killing assay

Peripheral Blood Mononuclear Cells (PBMC) were separated from the blood of healthy blood donors. PBMCs were cultured in ImmunoCult-XF T cell expansion medium (Stemcell Technologies, 10981) for 10 days, with the addition of ImmunoCult Human CD3/CD28/CD2 T Cell Activator (first three days, 25 μL/mL, Stemcell Technologies) and IL-2 (10 ng/mL, Pepro Tech, 10 days) to obtain activated T cells. Tumor cells were pretreated with butyrate (4 mM) for 48 hours, digested with trypsin, and inoculated into a 24-well plate overnight. Tumor cells were co-cultured with activated T cells in a 10:1 ratio in DMEM/F12 medium containing CD3 antibodies (100 ng/mL, eBioscience, Thermo Scientific) and IL-2 (10 ng/mL) for 48 hours. The surviving tumor cells were fixed in methanol for 10 minutes, then stained with 0.1% crystal violet and photographed. After washing with 33% (v/v) acetic acid solution, the optical density (OD) value was measured at 570 nm.

## Supplementary Material

Supplementary figures and table.

## Data Availability

All data supporting the findings of this study are available from the corresponding author (Yanping Xu, yanpingxu@tongji.edu.cn) upon request.
