# Supplementary material for: Butyrate enhances tumor immune evasion by increasing PD-L1 abundance
Source: Int J Biol Sci. 2026 Jul 20;22(12):6752–66. doi: 10.7150/ijbs.131184 (PMC13412482; doi:10.7150/ijbs.131184)
Supplement: Supplementary file 1 — Supplementary figures and table. [file ijbsv22p6752s1.pdf]

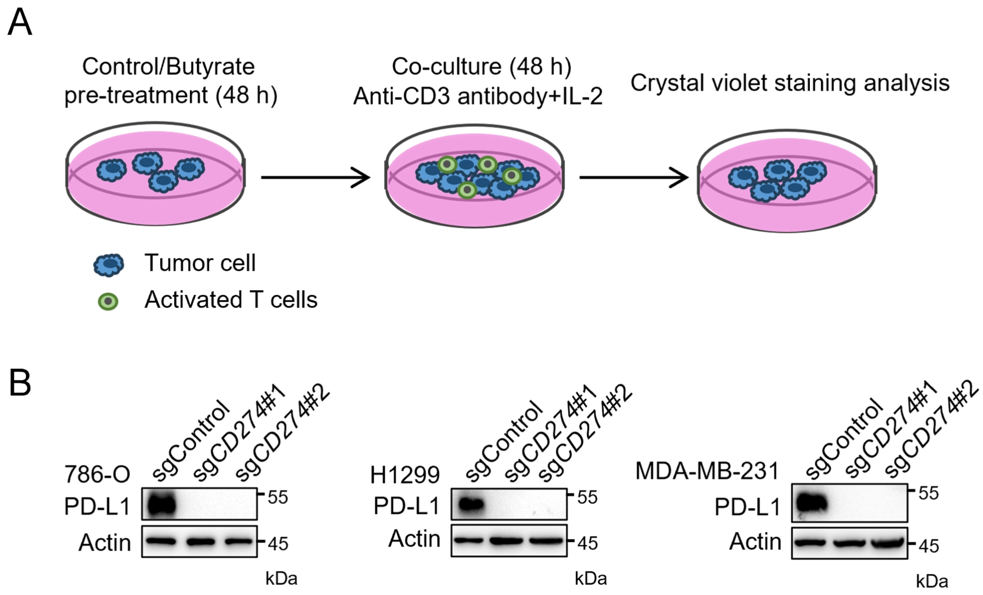

Fig. S1 Schematic diagram of T cell-mediated tumor cell killing and construction of *CD274* KO cell line

(**A**) Schematic diagram of T cell-mediated tumor cell killing assay. (**B**) Western blot analysis detected the efficiency of knocking out *CD274* in 786-O, H1299, and MDA-MB-231 cells.

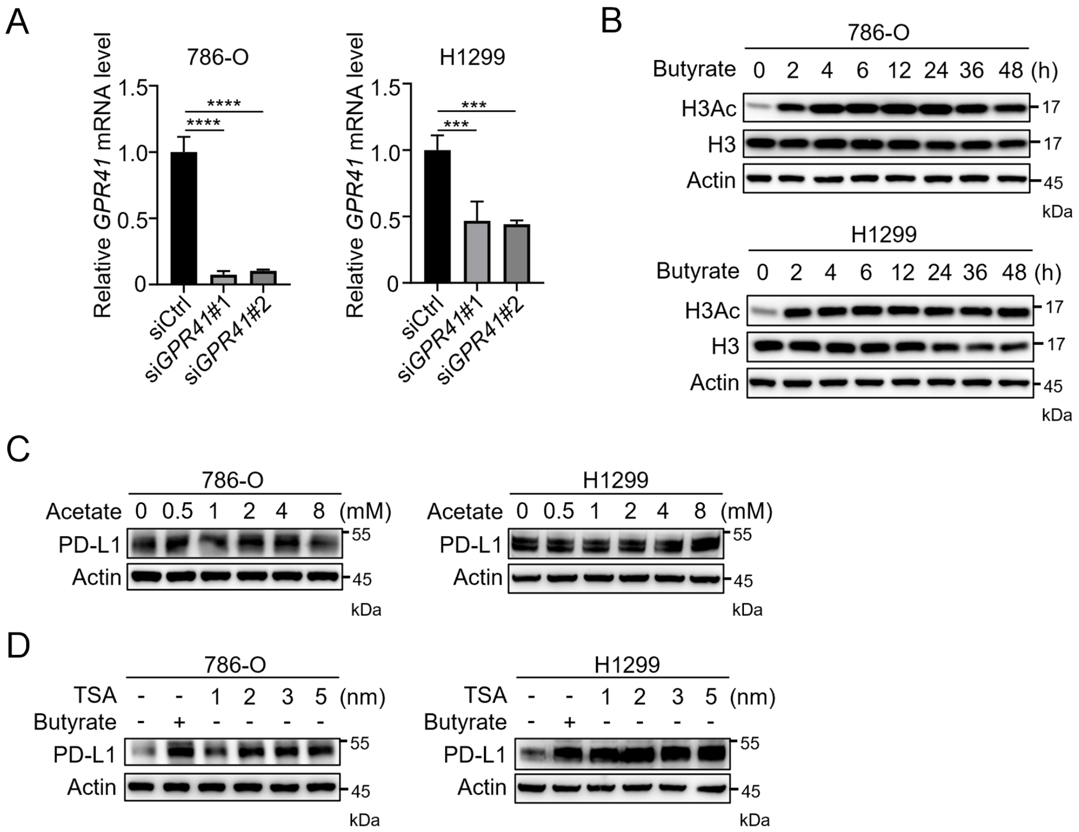

Fig. S2 Butyrate upregulates PD-L1 through HDAC inhibition-dependent epigenetic remodeling

(**A**) qRT-PCR analysis of *GPR41* levels in 786-O and H1299 cells transfected with siRNAs-targeting control or *GPR41* for 48 h. Values are means ± SD from n = 3 independent experiments. Statistical differences were determined by ordinary one-way ANOVA. ***P < 0.001, ****P < 0.0001. (**B**) Western blot analysis of histone H3 and H3 acetylation (H3Ac) levels in 786-O and H1299 cells treated with 4 mM butyrate for different times as indicated. (**C**) Western blot analysis of PD-L1 protein levels in 786-O and H1299 cells treated with different concentrations of acetate as indicated for 48 h. (**D**) Western blot analysis of PD-L1 protein levels in 786-O and H1299 cells treated with butyrate (4 mM) or TSA for 24 h.

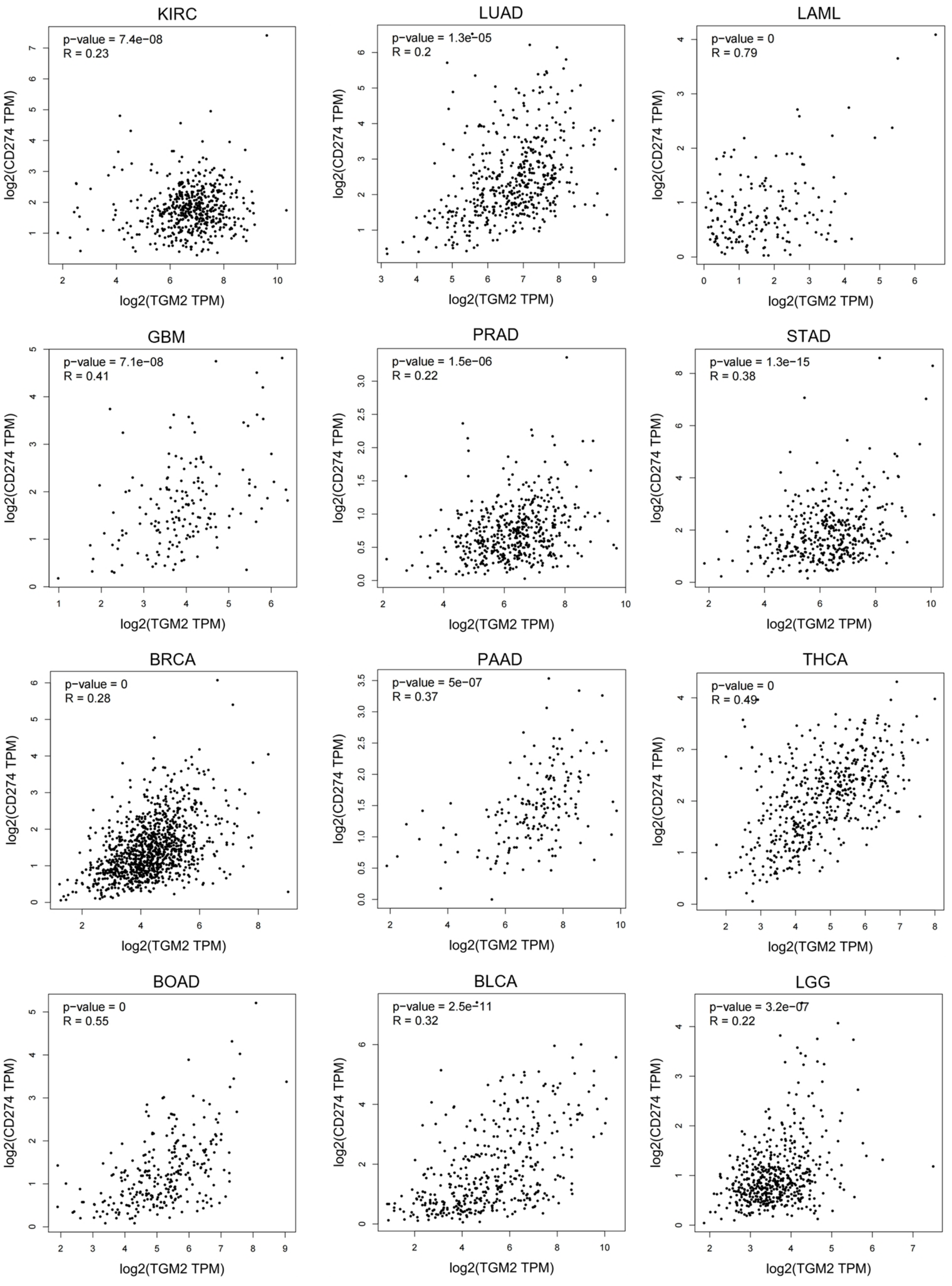

Fig. S3 Analyze the correlation between PD-L1 and TGM2 in multiple cancers from the TCGA database

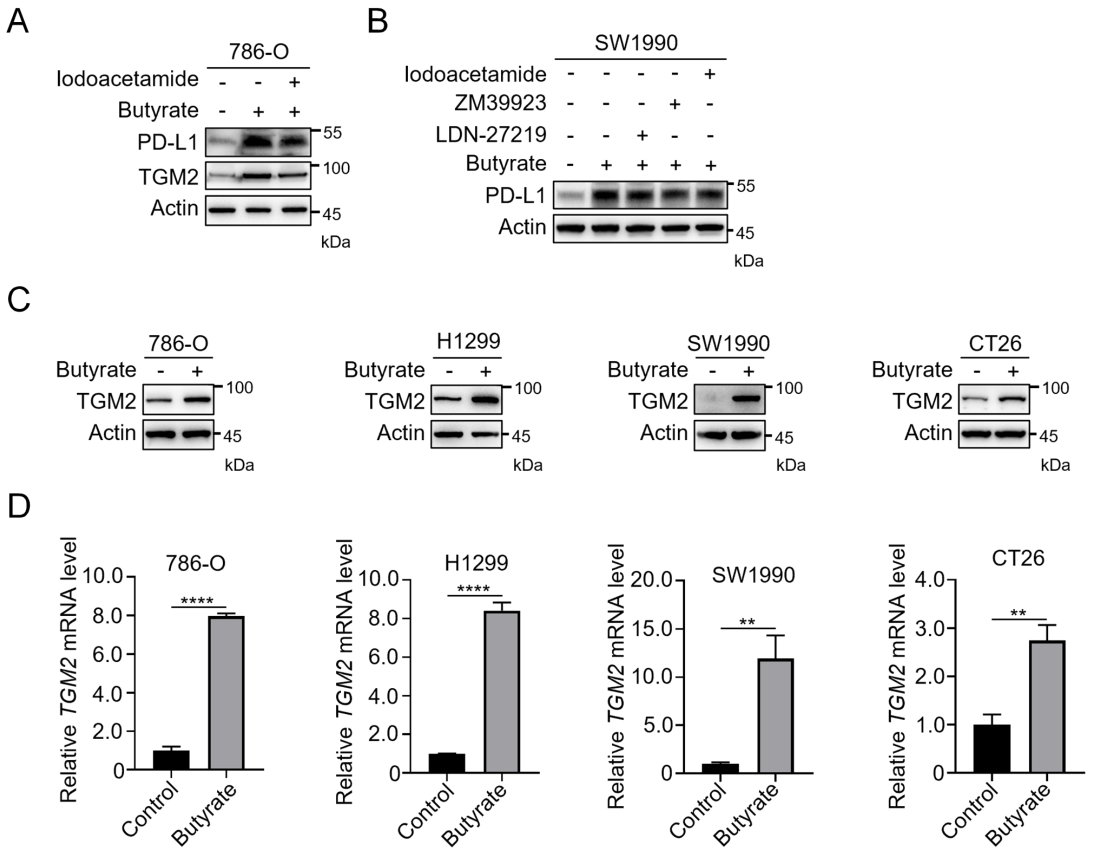

Fig. S4 TGM2 contributes to butyrate-induced PD-L1 upregulation via a distinct regulatory pathway

(**A**) Western blot analysis of PD-L1 and TGM2 protein levels in 786-O and H1299 cells treated with butyrate (4 mM, 48 h) or iodoacetamide (5 µM, 54 h). (**B**) Western blot analysis of PD-L1 protein levels in SW1990 cells treated with butyrate (4 mM, 48 h), ZM39923 (20 nM, 54 h), LDN-27219 (5 µM, 54 h), or iodoacetamide (5 µM, 54 h). (**C**) Western blot analysis of TGM2 protein levels in 786-O, H1299, SW1990, and CT26 cells treated with butyrate (4 mM, 48 h). (**D**) qRT-PCR analysis of *TGM2* levels in 786-O, H1299, SW1990, and CT26 cells treated with butyrate (4 mM, 48 h). Values are means ± SD from n = 3 independent experiments. Statistical differences were determined by Student's *t* test. $^{**}P < 0.01$, $^{****}P < 0.0001$.

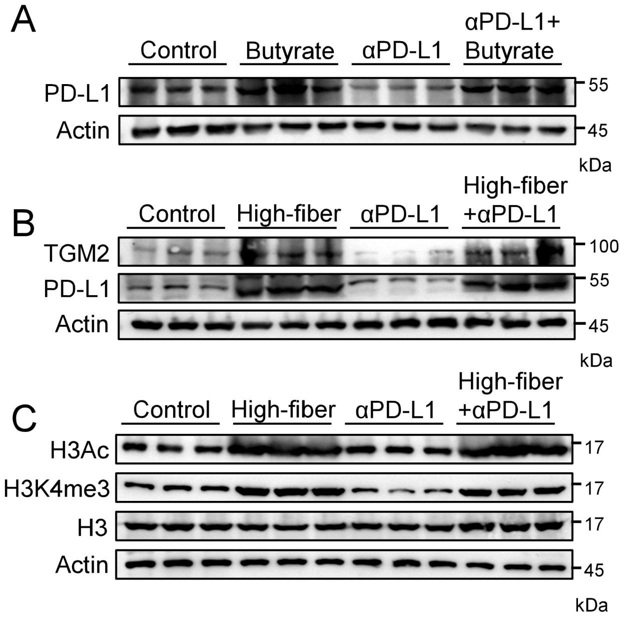

Fig. S5 Detection of protein levels in tumor tissue

(**A**) Western blot analysis of PD-L1 protein levels in tumor tissues from the CT26 subcutaneous model under different treatment conditions. (**B**) Western blot analysis of PD-L1 and TGM2 protein levels in tumor tissues from the AOM-DSS model under different dietary and treatment conditions. (**C**) Western blot analysis of histone H3Ac and H3K4me3 levels in tumor tissues from the AOM-DSS model, with total histone H3 and Actin as loading controls.

Table 1

Dyets#D210617 Control			
Ingredient	kcal/g	gram	kcal
Caisein	4	200	800
L-Cystine	4	3	12
Sucrose	4	66.8	267.2
Cornstarch	4	449.4	1798
Dyetrose	4	117.5	470
Soybean Oil	9	25	225
Lard	9	45	405
Cellulose	0	50	0
Mineral Mix	0	35	0
Vitamin Mix	4	10	40
Choline Bitartrate	0	2.5	0
Blue Dye	0	0.05	0
	gm%	kcal%	
Protein	20.2	20.2	
Carbohydrate	64.1	64.1	
Fat	7	15.7	

Dyets#D210616 High fiber			
Ingredient	kcal/g	gram	kcal
Caisein	4	200	800
L-Cystine	4	3	12
Sucrose	4	66.8	267.2
Cornstarch	4	449.4	1798
Dyetrose	4	55.6	222.4
Inulin	1.05	235.6	247.38
Soybean Oil	9	25	225
Lard	9	45	405
Cellulose	0	50	0
Mineral Mix	0	35	0
Vitamin Mix	4	10	40
Choline Bitartrate	0	2.5	0
Red Dye	0	0.05	0
	gm%	kcal%	
Protein	17.2	20.2	
Carbohydrate	69.4	64.1	
Fat	5.9	15.7	
